# Remote ischemic preconditioning reduces contrast-induced nephropathy in patients undergoing coronary angiography or percutaneous coronary intervention: an updated meta-analysis of randomized controlled trials

**DOI:** 10.3389/fcvm.2026.1916701

**Published:** 2026-09-18

**Authors:** Qingyi Yang, Yi Guan, Yanling Zhang, Zhenyu Zhou

**Affiliations:** Department of Cardiology, The Second Clinical Medical College of North Sichuan Medical College, Nanchong, China

**Keywords:** remote ischemic preconditioning, contrast-induced nephropathy, coronary angiography, percutaneous coronary intervention, meta-analysis

## Abstract

**Background:**

Contrast-induced nephropathy (CIN) is a common complication following coronary angiography (CA) or percutaneous coronary intervention (PCI) and is associated with prolonged hospitalization and adverse clinical outcomes. Remote ischemic preconditioning (RIPC), a noninvasive and nonpharmacological intervention, has been proposed as a potential strategy for preventing CIN. However, evidence from randomized controlled trials (RCTs) remains inconsistent.

**Methods:**

This meta-analysis of RCTs aimed to evaluate the efficacy of RIPC for preventing CIN in patients undergoing CA or PCI. The primary outcome was the incidence of CIN. For dichotomous outcomes, risk ratios (RRs) with 95% confidence intervals (CIs) were pooled using the Mantel–Haenszel fixed-effect model. For continuous outcomes, standardized mean differences (SMDs) with 95% CIs were pooled using a fixed-effect model when heterogeneity was low and a random-effects model when substantial heterogeneity was present. Trial sequential analysis was performed for dichotomous outcomes to estimate the robustness of the cumulative evidence and the required information size. This study was prospectively registered with PROSPERO (CRD420251269657).

**Results:**

Twenty-six RCTs including 4,169 participants were included. Overall, RIPC significantly reduced the risk of CIN compared with the control treatment (RR 0.49, 95% CI 0.41–0.59, *P* < 0.00001; *I*^2^ = 0%). Regarding renal function, RIPC significantly attenuated postprocedural increases in serum creatinine levels (SMD −1.00, 95% CI −1.60 to −0.39, *P* = 0.001) and was associated with lower postprocedural serum creatinine levels (SMD −0.23, 95% CI −0.39 to −0.08, *P* = 0.003). RIPC was also associated with a lower risk of major adverse events (MAEs) (RR 0.55, 95% CI 0.41–0.75, *P* = 0.0001). No significant differences were observed in postprocedural eGFR, neutrophil gelatinase-associated lipocalin (NGAL), cystatin C, all-cause mortality, dialysis requirement, or rehospitalization.

**Conclusion:**

In patients undergoing coronary angiography or percutaneous coronary intervention, RIPC was associated with reduced risks of CIN and MAEs, attenuated postprocedural increases in serum creatinine levels, and lower postprocedural serum creatinine levels, while evidence for postprocedural eGFR, NGAL, cystatin C, and clinical outcomes remains inconclusive.

**Systematic Review Registration:**

https://www.crd.york.ac.uk/PROSPERO/view/CRD420251269657, PROSPERO CRD420251269657.

## Introduction

Contrast-induced nephropathy (CIN) is a common complication following coronary angiography (CA) or percutaneous coronary intervention (PCI) and is associated with prolonged hospitalization and adverse clinical outcomes (1–3). The European Society of Urogenital Radiology (ESUR) formally defines CIN as an increase in serum creatinine of ≥0.5 mg/dL (44.2 μmol/L) or a relative elevation of >25% from the baseline value, occurring within 48–72 h following contrast medium (CM) administration (4). The Kidney Disease: Improving Global Outcomes (KDIGO) criteria define acute kidney injury (AKI) as an increase in serum creatinine of ≥0.3 mg/dL within 48 h or an increase to ≥1.5 times baseline within 7 days (5). The incidence of CIN varies widely, from less than 1% in low-risk populations to 20%–50% in high-risk subgroups, with PCI generally conferring a higher risk than diagnostic angiography alone (6). Well-recognized risk factors include chronic kidney disease, diabetes mellitus, advanced age, and volume depletion (7, 8). Although current guidelines recommend preventive strategies for at-risk patients, conventional approaches such as hydration are not fully protective, particularly in moderate-to-high-risk patients, underscoring the need for additional renoprotective interventions (9, 10).

Remote ischemic preconditioning (RIPC) is a noninvasive, nonpharmacological intervention that activates endogenous protective mechanisms through brief cycles of limb ischemia and reperfusion (11, 12). Accumulating evidence indicates that RIPC may mitigate ischemia-reperfusion injury, a mechanism implicated in contrast-induced renal injury (13, 14). Nevertheless, clinical trials evaluating RIPC for CIN prevention have yielded conflicting results, including trials involving high-risk patients undergoing CA or PCI. Moreover, the patient populations most likely to benefit remain incompletely defined.

Previous meta-analyses have suggested a potential renoprotective effect of RIPC; however, their conclusions were limited by small sample sizes, heterogeneous populations, and the absence of trial sequential analysis (15, 16). Given the expanding body of randomized evidence and the persistent uncertainty regarding the clinical settings in which RIPC is most effective, an updated systematic review and meta-analysis is warranted. Therefore, we conducted an updated systematic review and meta-analysis of randomized controlled trials to evaluate the efficacy and safety of RIPC for preventing CIN after CA or PCI. We further examined renal function changes, major adverse events, and clinically relevant subgroups, and applied trial sequential analysis to assess the robustness and conclusiveness of the cumulative evidence.

## Methods

### Data sources and search strategies

This systematic review and meta-analysis was conducted and reported in accordance with the Preferred Reporting Items for Systematic Reviews and Meta-Analyses (PRISMA) guidelines (17). The protocol was registered in PROSPERO (CRD420251269657). A comprehensive literature search was performed in PubMed, Embase, and the Cochrane Central Register of Controlled Trials via the Cochrane Library from database inception to March 1, 2026, without language restrictions. The search strategy combined controlled vocabulary and free-text terms related to “ischemic preconditioning”, “remote ischemic precondition”, “RIPC”, “acute kidney injury”, “contrast associated acute kidney injury”, “contrast induced nephropathy”, “contrast-induced acute kidney injury”, “CIN”, and “randomized controlled trial”. The detailed search strategy is provided in Supplementary Table S1. In addition, the reference lists of all included studies and relevant reviews were manually screened to identify additional eligible studies.

### Inclusion and exclusion criteria

After removal of duplicates, the selection process comprised two stages: (1) initial screening of titles and abstracts to exclude clearly irrelevant studies and (2) full-text assessment of potentially eligible articles. Two reviewers independently evaluated study eligibility, and discrepancies were resolved through discussion or consultation with a third reviewer. Studies were included if they met all of the following criteria: (1) randomized controlled trial (RCT) design; (2) enrolled patients undergoing CA or PCI; (3) RIPC with a sham procedure, no RIPC, standard care, or usual care; and (4) reported at least one relevant outcome, including CIN, serum creatinine, cystatin C, neutrophil gelatinase-associated lipocalin (NGAL), estimated glomerular filtration rate (eGFR), MAEs, all-cause mortality, dialysis requirement, or rehospitalization. Studies were excluded if they were nonrandomized studies, observational studies, animal or experimental studies, duplicate publications, or non-original articles, such as reviews, editorials, and commentaries. During study selection and data extraction, trials originating from the same research groups or institutions were further assessed for potential patient overlap by comparing study sites, recruitment periods, eligibility criteria, procedural settings, sample sizes, baseline characteristics, and trial registration information where available. We carefully assessed these studies for potential overlap by comparing the study sites, recruitment periods, eligibility criteria, and enrolled patient populations, and found no evidence of duplicated patient cohorts. Studies involving ischemic postconditioning rather than RIPC were also excluded. Studies without a valid control group or extractable outcome data were excluded. Non-English studies were translated when necessary.

### Data extraction and quality assessment

Two reviewers independently extracted data using a predefined data extraction form. Extracted data included study characteristics, baseline patient characteristics, procedural details, RIPC and control protocols, CIN definitions, contrast media dose, hydration protocols (The completeness and standardization of reported hydration protocols were further summarized in Supplementary Table S4), and all prespecified outcomes. Discrepancies were resolved through discussion or consultation with a third senior reviewer. Two reviewers independently assessed the risk of bias using the Cochrane Risk of Bias 2 (RoB 2) tool, with each study categorized as having low risk of bias, some concerns, or high risk of bias. The certainty of evidence for key outcomes was evaluated using the Grading of Recommendations Assessment, Development and Evaluation (GRADE) framework, with evidence rated as high, moderate, low, or very low.

### Statistical analysis

Data synthesis was performed using Review Manager software, version 5.4 (RevMan 5.4; The Cochrane Collaboration). For dichotomous outcomes, including CIN incidence, MAEs, all-cause mortality, dialysis requirement, and rehospitalization, effect estimates were expressed as RRs with 95% CIs. For continuous outcomes, including serum creatinine, cystatin C, NGAL, and eGFR, standardized mean differences (SMDs) with 95% CIs were calculated because different scales, units, measurement methods, or reporting formats were used across studies. Statistical heterogeneity was assessed using Cochran's *Q-*test, with *P* < 0.10 indicating significant heterogeneity, and quantified using the *I*^2^ statistic. Heterogeneity was interpreted as low, moderate, or high when *I*^2^ values were <25%, 25%–50%, or >50%, respectively. A fixed-effect model was used when statistical heterogeneity was low, whereas a random-effects model was applied when moderate or high heterogeneity was present. For dichotomous outcomes, the Mantel–Haenszel method was used, whereas for continuous outcomes, the inverse-variance method was applied. Results from analyses with high heterogeneity were interpreted with caution because of potential between-study variability. Prespecified subgroup analyses were conducted according to baseline eGFR (<60 vs. ≥60 mL/min/1.73 m^2^), type of procedure, presence of diabetes mellitus, timing of outcome assessment (<48 vs. ≥48 h after the procedure), CIN definition (ESUR-compatible vs. KDIGO-type vs. Other), number of RIPC cycles (3 vs. 4 cycles), and cuff pressure strategy [200 mmHg vs. systolic blood pressure (SBP) + 50 mmHg], when sufficient data were available. For CIN, studies were classified according to the principal serum creatinine threshold reported in the original trials (Supplementary Table S3); the post-contrast assessment window was recorded separately but was not used as the primary basis for categorization, and the primary meta-analysis retained the outcome definition used in each study. An exploratory subgroup analysis of MAEs according to baseline eGFR (<60 vs. ≥60 mL/min/1.73 m^2^) was also performed. A sensitivity analysis restricted to studies using ESUR-compatible definitions of CIN was also performed. In addition, because the trial by Jia et al. was the only included study evaluating a delayed-window RIPC strategy (21), we performed a sensitivity analysis excluding this study to assess whether the overall pooled effect was driven by the delayed-window RIPC protocol. Publication bias was visually assessed using funnel plots when at least 10 studies were available for an outcome. For outcomes with at least 10 included studies, Egger's regression test was further performed to assess potential small-study effects, with *P* < 0.10 considered suggestive of funnel plot asymmetry. A two-sided *P* value <0.05 was considered statistically significant, except for heterogeneity testing, for which *P* < 0.10 was used. TSA was performed for CIN, MAEs, all-cause mortality, dialysis requirement, and rehospitalization using TSA software version 0.9.5.10. The required information size was estimated with a two-sided type I error of 0.05, power of 80%, control event proportion, anticipated relative risk reduction, and heterogeneity adjustment. Evidence was considered conclusive when the cumulative Z-curve crossed the TSA monitoring boundary or reached the required information size.

## Results

### Study selection and characteristics

The study selection process is illustrated in Figure 1. A total of 898 records were retrieved from PubMed, Embase, and the Cochrane Library. After duplicate removal and title/abstract screening, 73 articles were retrieved for full-text evaluation; ultimately, 26 randomized controlled trials (RCTs) satisfied all predefined inclusion criteria (14, 18–42). No evidence of overlapping patient populations was identified among trials originating from the same research groups or institutions. Risk-of-bias evaluations for the 26 included RCTs are displayed in Supplementary Figure S1. Baseline characteristics of the enrolled trials and participant populations are summarized in Table 1. Overall, demographic and clinical variables were generally comparable between the RIPC and control groups across all trials. The certainty of evidence for each primary and secondary outcome was evaluated via the GRADE approach, with detailed ratings provided in Supplementary Table S2.

**Figure 1:**
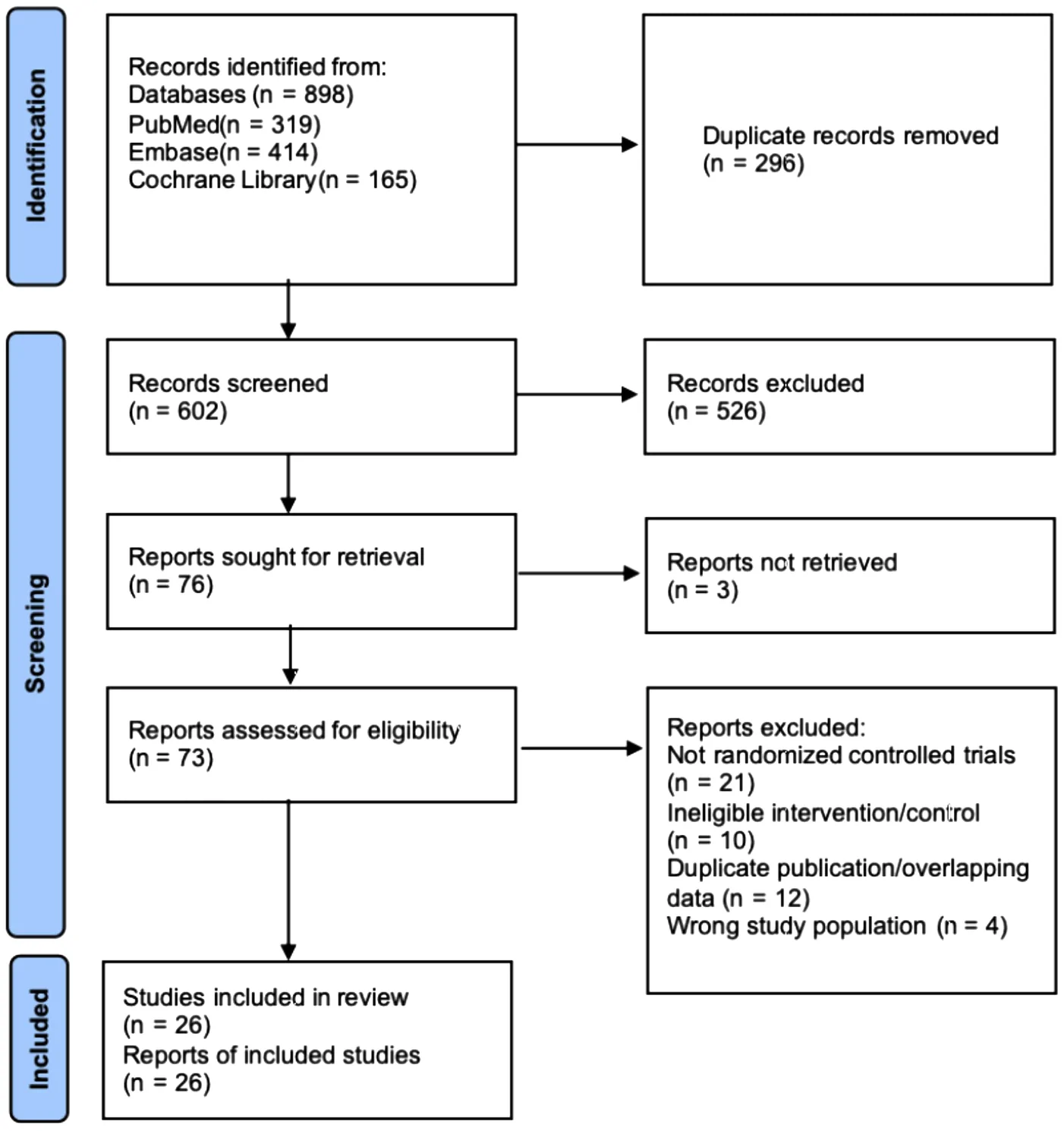
Flow diagram of study selection.

**Table 1:** Baseline characteristics of included studies.

Study	Blinding	Inclusion criteria	Number involved, R/C	Sex, M/F	Age, years	eGFR, mL/min/1.73 m2	Serum creatinine, mg/dL	Contrast agent dosage, mL	Time-to-procedure	RIPC application	Definition of CIN
Hoole et al. (31)	Single-blind	PCI	R:104 C:98	R: 84/20 C: 74/24	R: 63.2 ± 10.1 C: 61.8 ± 10.3	–	–	R: 196.7 ± 80.1 C: 187.5 ± 74.2	<1 h before the PCI	5 min inflations of a blood pressure cuff to 200 mm Hg around the upper arm, followed by 5 min intervals of deflations, repeated 3 times	An increase in Scr >25% from the baseline at 24 h after PCI
Singh et al. (18)	Double-blind	PCI	R:51 C:51	R: 23/28 C: 25/26	R: 67.8 ± 7.6 C: 69.0 ± 8.6	R: 48.5 ± 12.0 C: 46.6 ± 10.2	R: 1.42 ± 0.58 C: 1.41 ± 0.34	R: 197.5 ± 114.3 C: 196.3 ± 118.8	<30 min before the PCI	5 min inflations of a blood pressure cuff to 200 mm Hg around the upper arm, followed by 5 min intervals of deflations, repeated 3 times	An increase in Scr ≥25% and/or an Scr rise ≥0.5 mg/dL from the baseline within 48 h after PCI
Xu et al. (19)	Single-blind	PCI	R:102 C:98	R: 68/34 C: 68/30	R: 69.1 ± 3.8 C: 68.9 ± 2.9	R: 99.1 ± 20.6 C: 100.8 ± 28.2	R: 0.88 ± 0.19 C: 0.84 ± 0.20	R: 171.8 ± 37.9 C: 163.3 ± 39.0	<2 h before the PCI	5 min inflations of a blood pressure cuff to 200 mm Hg around the upper arm, followed by 5 min intervals of deflations, repeated 3 times	An increase in Scr ≥25% and/or an Scr rise ≥0.5 mg/dL from the baseline at 16 h after PCI
Luo et al. (27)	Single-blind	PCI	R:101 C:104	R: 78/23 C: 78/26	R: 59.2 ± 10.3 C: 59.3 ± 9.5	R: 101 ± 20 C: 100 ± 20	–	R: 154.0 ± 46.0 C: 145.0 ± 41.0	<2 h before the PCI	5 min inflations of a blood pressure cuff to 200 mm Hg around the upper arm, followed by 5 min intervals of deflations, repeated 3 times	An increase in Scr ≥25% and/or an Scr rise ≥0.5 mg/dL from the baseline at 16 h after PCI
Er et al. (14)	-	CA	R:50 C:50	R: 34/16 C: 37/13	R: 73.2 ± 9.1 C: 72.7 ± 11.4	R: 40.6 ± 8.7 C: 41.3 ± 11.9	R: 1.63 (1.47,1.81) C: 1.62 (1.39,1.93)	R: 124.0 ± 44.0 C: 103.0 ± 41.0	<45 min before the CA	5 min inflations of a blood pressure cuff to 200 mm Hg around the upper arm, followed by 5 min intervals of deflations, repeated 4 times	An increase in Scr ≥25% and/or an Scr rise ≥0.5 mg/dL from the baseline at 48 h after CA
Igarashi et al. (29)	-	CA or PCI	R:30 C:30	R: 20/10 C: 23/7	R: 71.3 ± 8.1 C: 70.8 ± 7.6	R: 47.4 ± 9.4 C: 48.9 ± 6.0	R: 1.15 ± 0.29 C: 1.12 ± 0.17	R: 92.9 ± 33.2 C: 91.8 ± 39.4	<2 h before the PCI or CA	5 min inflations of a blood pressure cuff to 200 mm Hg around the upper arm, followed by 5 min intervals of deflations, repeated 4 times	An increase in Scr ≥25% and/or an Scr rise ≥0.5 mg/dL from the baseline within 48 h after CA or PCI
Savaj et al. (23)	–	CA	R:48 C:48	R: 17/31 C: 14/34	R: 63.0 ± 8.9 C: 60.9 ± 9.6	R: 78.2 ± 25.8 C: 94.7 ± 40.2	R: 1.3 ± 0.4 C: 1.1 ± 0.3	R: 126.6 ± 77.2 C: 123.8 ± 66.6	<15 min before the CA	5 min inflations of a blood pressure cuff to 200 mm Hg around the upper arm, followed by 5 min intervals of deflations, repeated 3 times	An increase in Scr ≥30% and/or an Scr rise ≥0.3 mg/dL from the baseline at 24 h after CA
Yamanaka et al. (26)	Single-blind	PCI	R:47 C:47	R: 34/13 C: 36/11	R: 67.0 ± 12.0 C: 67.0 ± 15.0	R: 73.0 ± 20.0 C: 79.0 ± 33.0	R: 0.82 ± 0.21 C: 0.87 ± 0.44	R: 177.0 ± 53.0 C: 199.0 ± 87.0	–	5 min inflations of a blood pressure cuff to 200 mm Hg around the upper arm, followed by 5 min intervals of deflations, repeated 3 times	An increase in Scr ≥25% and/or an Scr rise ≥0.5 mg/dL from the baseline within 48–72 h after PCI
Zagidullin et al. (42)	–	CA	R: 26 C: 25	R: 21/5 C: 22/3	R: 60.5 ± 1.95 C: 62.96 ± 1.72	–	R: 1.41 ± 0.13 C: 1.21 ± 0.11	R: 155.8 ± 16.9 C: 148.3 ± 16.7	<1 h before the CA	5 min inflations of a blood pressure cuff to level 50 mm Hg above SBP around the upper arm, followed by 5 min intervals of deflations, repeated 3 times	An increase in Scr ≥25% and/or an Scr rise ≥0.5 mg/dL from the baseline at 48 h after CA
Zhou et al. (35)	–	PCI	R: 50 C: 57	R: 30/20 C: 35/22	R: 69.42 ± 7.07 C: 69.14 ± 7.80	R: 69.99 ± 8.91 C: 68.02 ± 10.17	R: 1.02 ± 0.20 C: 1.06 ± 0.22	R: 114.76 ± 44.22 C: 108.82 ± 43.25	<1 h before the PCI	5 min inflations of a blood pressure cuff to 200 mm Hg around the upper arm, followed by 5 min intervals of deflations, repeated 4 times	An increase in Scr ≥25% and/or an Scr rise ≥0.5 mg/dL from the baseline within 48–72 h after PCI
Ghaemian et al. (36)	Double-blind	CA or PCI	R: 66 C: 66	R: 51/15 C: 42/24	R: 66.15 ± 8.63 C: 65.27 ± 8.9	R: 41.86 ± 8.16 C: 42.77 ± 9.46	R: 1.5 (1.4–1.73) C: 1.5 (1.4–1.6)	R: 90 (80–112.5) C: 100 (80–116)	<40 min before The PCI or CA	5 min inflations of a blood pressure cuff to level 50 mm Hg above SBP around the upper arm, followed by 5 min intervals of deflations, repeated 4 times	An Scr rise ≥0.3 mg/dL from the baseline within 48–72 h after CA or PCI
Moretti et al. (30)	Double-blind	PCI	R: 107 C: 116	R: 74/33 C: 80/36	R: 71.8 ± 10.6 C: 72.6 ± 9.6	–	–	R: 171.4 ± 78.6 C: 174.4 ± 83.7	<45 min before the PCI	5 min inflations of a blood pressure cuff to 200 mm Hg around the upper arm, followed by 5 min intervals of deflations, repeated 4 times	An increase in Scr ≥25% and/or an Scr rise ≥0.5 mg/dL from the baseline within 48 h after PCI
Valappil et al. (41)	Single-blind	PCI	R: 50 C: 50	R: 43/7 C: 40/10	R: 62.8 ± 9.1 C: 60.5 ± 8.5	R: 44.3 (39.7-49.7) C: 46.3 (40.2-53.1)	R: 1.5 (1.3–1.7) C: 1.4 (1.4–1.6)	–	<45 min before the PCI	5 min inflations of a blood pressure cuff to level 50 mm Hg above SBP around the upper arm, followed by 5 min intervals of deflations, repeated 4 times	An increase in Scr ≥25% and/or an Scr rise ≥0.5 mg/dL from the baseline within 24–48 h after PCI
Gholoobi et al. (24)	Double-blind	CA	R: 25 C: 26	R: 18/7 C: 12/14	R: 67.08 ± 12.49 C: 70.31 ± 11.18	–	–	–	<1 h before the CA	5 min inflations of a blood pressure cuff to level 50 mm Hg above SBP around the upper arm, followed by 5 min intervals of deflations, repeated 4 times	An Scr rise ≥0.3 mg/dL from the baseline within 48 h after CA
Wojciechowska et al. (28)	–	PCI	R: 62 C: 61	R: 42/20 C: 42/19	R: 64.4 ± 10.5 C: 62.9 ± 8.60	R: 105.6 ± 36.3 C: 107.3 ± 36.5	R: 0.86 ± 0.27 C: 0.86 ± 0.26	R: 155.5 ± 76.7 C: 143.7 ± 55.7	<2 h before the PCI	5 min inflations of a blood pressure cuff to 200 mm Hg around the upper arm, followed by 5 min intervals of deflations, repeated 3 times	An increase in Scr ≥25% and/or an Scr rise ≥0.5 mg/dL from the baseline within 24–72 h after contrast administration
Roubille et al. (34)	Single-blind	CA or PCI	R: 98 C: 104	R: 68/30 C: 72/32	R: 75.6 ± 9.2 C: 76.7 ± 7.6	R: 43 ± 11 C: 42 ± 10	R: 1.69 ± 0.57 C: 1.66 ± 0.50	R: 73 (45–121) C: 80 (50–111)	–	5 min inflations of a blood pressure cuff to 200 mm Hg around the upper arm, followed by 5 min intervals of deflations, repeated 4 times	An increase in Scr ≥25% and/or an Scr rise ≥0.5 mg/dL from the baseline within 48–72 h after CA or PCI
Yan et al. (32)	–	PCI	R: 142 C: 140	R: 110/32 C: 103/37	R: 65 ± 10 C: 65 ± 12	R:66 ± 21 C: 65 ± 20	R: 1.12 ± 0.45 C: 1.09 ± 0.32	R: 223 ± 58 C:217 ± 61	<30 min before the PCI	5 min inflations of a blood pressure cuff to 200 mm Hg around the upper arm, followed by 5 min intervals of deflations, repeated 3 times	An increase in Scr ≥25% and/or an Scr rise ≥0.5 mg/dL from the baseline within 48–72 h after PCI
Stokfisz et al. (33)	–	PCI	R: 50 C: 51	66/35	R: 66 (59–70) C: 65 (60–74)	R:81.6 (62.5–86.1) C: 76.8 (59.7–85.0)	R: 0.98 (0.92–1.09) C: 1.00 (0.93–1.14)	R:100 (80–140) C:110 (90–140)	<1 h before the PCI	5 min inflations of a blood pressure cuff to 200 mm Hg around the upper arm, followed by 5 min intervals of deflations, repeated 4 times	An increase in Scr ≥25% and/or an Scr rise ≥0.5 mg/dL from the baseline within 48–72 h after contrast exposure
Sahu et al. (39)	Single-blind	CA	R: 45 C: 42	R: 38/7 C: 30/12	R: 57.76 ± 7.8 C: 55.35 ± 10.76	R:34.0 ± 8.19 C: 33.24 ± 7.25	R: 2.16 ± 0.46 C: 2.14 ± 0.44	R:40.02 ± 7.61 C:37.04 ± 6.04	<45 min before the CA	5 min inflations of a blood pressure cuff to level 50 mm Hg above SBP around the upper arm, followed by 5 min intervals of deflations, repeated 4 times	An increase in Scr ≥25% and/or an Scr rise ≥0.5 mg/dL from the baseline within 48 h after contrast medium administration
Jia et al. (21)	–	CA or PCI	R: 250 C: 251	R: 178/72 C:181/70	R: 72 (66–78) C: 76 (67–79)	R:69 (52–88) C:66 (52–87)	R:1.03 (0.97–1.35) C: 1.01 (0.80–1.32)	R:110 (60–150) C: 110 (60–150)	< 24 h before the PCI or CA	5 min inflations of a blood pressure cuff to 200 mm Hg around the upper arm, followed by 5 min intervals of deflations, repeated 4 times	an increase in serum creatinine of ≥0.3 mg/dL (or 26.5 μmol/L) from baseline within 48 h after CA or PCI
Otsuka et al. (20)	–	PCI	R: 69 C: 74	R: 53/16 C: 51/23	R: 70 ± 10 C: 72 ± 9	R:63 ± 15 C: 64 ± 17	R: 0.90 ± 0.21 C: 0.87 ± 0.22	R:105 (80–121) C:102 (70–130)	<1 h before the PCI	5 min inflation of a blood pressure cuff to 200 mmHg around the upper arm, followed by 5 min intervals of deflations, repeated 3 times	An increase in Scr ≥20% and/or an Scr rise ≥0.3 mg/dL from the baseline within 24 h after PCI
Guo et al. (40)	–	PCI	R: 110 C: 110	R: 68/42 C: 62/48	R: 71.69 ± 6.69 C: 70.81 ± 6.27	R:80.95 ± 25.38 C: 79.53 ± 27.00	R: 0.82 ± 0.20 C:0.85 ± 0.25	R:141 ± 59.5 C:143 ± 58.7	<2 h before the PCI	5 min inflations of a blood pressure cuff to 200 mm Hg around the upper arm, followed by 5 min intervals of deflations, repeated 3 times	An increase in Scr ≥25% and/or an Scr rise ≥0.5 mg/dL from the baseline within 48–72 h after contrast medium administration
Elserafy et al. (25)	–	PCI	R: 50 C: 50	R: 29/21 C: 29/21	R:65.16 ± 7.98 C:65.1 ± 7.07	R:45.81 ± 7.89 C:44.60 ± 7.72	R:1.68 ± 0.13 C:1.69 ± 0.12	R:125.20 ± 37.59 C:119.80 ± 38.41	<45 min before the PCI	5 min inflations of a blood pressure cuff to level 50 mmHg above SBP around the upper arm, followed by 5 min intervals of deflations, repeated 4 times	An increase in Scr ≥25% and/or an Scr rise ≥0.5 mg/dL from the baseline or creatinine clearance decreased >50% over the baseline level within 48 h after contrast medium administration
Bafna et al. (38)	Double-blind	CA	R: 81 C: 81	R: 53/28 C: 51/30	R:55.9 ± 8.3 C: 57.8 ± 8.0	R:51.76 ± 7.08 C:50.71 ± 7.65	R:1.42 ± 0.18 C:1.41 ± 0.18	–	<3 h before the CA	5 min inflations of a blood pressure cuff to 200 mm Hg around the upper arm, followed by 5 min intervals of deflations, repeated 4 times	An increase in Scr ≥25% and/or an Scr rise ≥0.5 mg/dL from the baseline within 48–72 h after contrast medium administration
Olafiranye et al. (22)	Double-blind	CA or PCI	R: 54 C: 55	R: 45/9 C: 47/8	R: 76.0 (71.0–81.8) C: 75.0 (71.0–79.5)	R:59.7 ± 23.9 C:62.1 ± 22.3	R: 1.3 (0.9–1.6) C: 1.2 (1.0–1.6)	R: 77.5 (45.0–147.5) C: 62.5 (50.0–110.0)	<4 h before the PCI or CA	5 min inflations of a blood pressure cuff to 200 mm Hg around the upper arm, followed by 5 min intervals of deflations, repeated 3 times	An increase in Scr ≥50% and/or an Scr rise ≥0.3 mg/dL from the baseline at 48 h after CA or PCI
Paramasivam et al. (37)	Double-blind	PCI	R: 211 C: 209	R: 148/63 C: 150/59	R: 65 (58–72.5) C: 63 (57–72)	R: 57 (46.5–78.5) C: 54 (42.0–71.0)	R: 1.2 (1.0, 1.5) C: 1.3 (1.0, 1.5)	R: 150 (130–180) C: 150 (130- 180)	<45 min before the PCI	5 min inflations of a blood pressure cuff to 200 mm Hg around the upper arm, followed by 5 min intervals of deflations, repeated 4 times	An increase in Scr ≥50% and/or an Scr rise ≥0.3 mg/dL from the baseline within 48 h after PCI

Data are presented as means ± SD or medians (quartiles Q1–Q3).

R, RIPC group; C, control group; CIN, contrast-induced nephropathy; PCI, percutaneous coronary intervention; CA, coronary angiography; eGFR, estimated glomerular filtration rate; RIPC, remote ischemic preconditioning; SBP, systolic blood pressure; –, not mentioned.

### Primary outcome

Data on the incidence of CIN were available in 25 of the 26 included trials, comprising 4,007 participants. Bafna et al. trial fulfilled the inclusion criteria but did not report extractable CIN event counts by treatment arm and was therefore not included in the primary CIN meta-analysis. The pooled analysis showed that RIPC significantly decreased the incidence of CIN compared with the control group (RR 0.49, 95% CI 0.41–0.59, *P* < 0.00001; *I*^2^ = 0%, P for heterogeneity = 0.75) (Figure 2).

**Figure 2:**
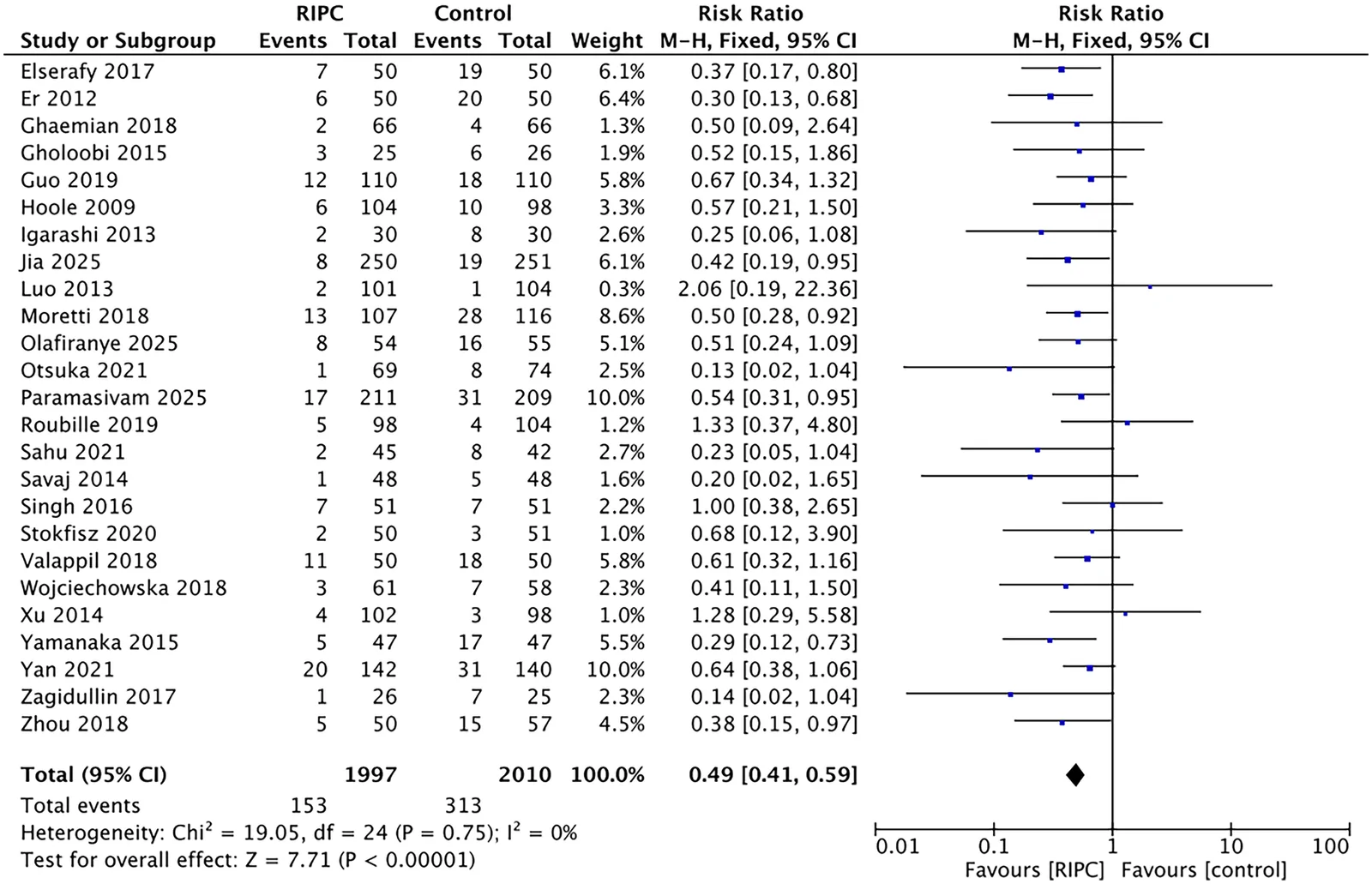
Effect of RIPC on the incidence of CIN.

Subgroup analyses were performed according to baseline renal function, diabetes status, procedural type, timing of post-procedure creatinine assessment, CIN definition, number of RIPC cycles, and cuff pressure strategy. In the subgroup analysis according to baseline renal function, RIPC significantly reduced the risk of CIN compared with the control group in patients with eGFR < 60 mL/min/1.73 m^2^ (RR 0.49, 95% CI 0.38–0.62, *P* < 0.00001), whereas no significant benefit was observed in patients with eGFR ≥ 60 mL/min/1.73 m^2^ (RR 0.82, 95% CI 0.36–1.85, *P* = 0.62) (Supplementary Figure S2A). In the subgroup analysis according to procedural type, RIPC significantly reduced the risk of CIN in PCI-only trials compared with the control group (RR 0.54, 95% CI 0.44–0.66, *P* < 0.00001). Similar results were observed in CA or mixed CA/PCI cohorts (RR 0.39, 95% CI 0.28–0.56, *P* < 0.00001) (Supplementary Figure S2B). The subgroup analysis by diabetes status revealed that in trials exclusively enrolling diabetic patients, RIPC was associated with a lower incidence of CIN compared with the control group (RR 0.50, 95% CI 0.28–0.89, *P* = 0.02). This was consistent with the results observed in mixed cohorts or studies without clear diabetes stratification (RR 0.51, 95% CI 0.42–0.62, *P* < 0.00001) (Supplementary Figure S2C). The subgroup analysis according to the timing of post-procedure creatinine assessment showed that RIPC significantly reduced the risk of CIN in studies assessing renal injury ≥48 h after catheterization compared with the control group (RR 0.49, 95% CI 0.40–0.59, *P* < 0.00001). This was consistent with the results observed in studies evaluating CIN < 48 h after the procedure (RR 0.52, 95% CI 0.35–0.77, *P* = 0.001) (Supplementary Figure S2D). When stratified by the number of RIPC cycles, both 3 cycles and 4 cycles protocols were associated with a significantly lower risk of CIN than control treatment, with pooled RRs of 0.53 (95% CI 0.41–0.70, *P* < 0.00001) and 0.46 (95% CI 0.36–0.59, *P* < 0.00001) (Supplementary Figure S2E). When stratified by cuff pressure strategy, RIPC was associated with a significantly lower risk of CIN in both studies using a fixed cuff pressure of 200 mmHg (RR 0.51, 95% CI 0.42–0.63, *P* < 0.00001) and those using SBP + 50 mmHg (RR 0.42, 95% CI 0.28–0.63, *P* < 0.0001) (Supplementary Figure S2F). In the subgroup analysis according to CIN definition, RIPC significantly reduced the risk of CIN across studies classified as ESUR-compatible (RR 0.52, 95% CI 0.42–0.65, *P* < 0.00001), KDIGO-type (RR 0.50, 95% CI 0.35–0.72, *P* = 0.0002), and Other (RR 0.28, 95% CI 0.14–0.57, *P* = 0.0003), (Supplementary Figure S2G). The pooled effect estimate remained robust in sensitivity analyses restricted to studies using ESUR-compatible definitions of CIN (RR 0.52, 95% CI 0.42–0.65; *I*^2^ = 0%; *P* < 0.00001) (Supplementary Figure S11). In addition, after exclusion of the Jia et al. trial, which was the only study evaluating a delayed-window RIPC strategy, the pooled effect remained essentially unchanged and statistically significant (RR 0.50, 95% CI 0.41–0.60; *I*^2^ = 0%; *P* < 0.00001) (Supplementary Figure S12).

Formal tests for subgroup differences showed no statistically significant interactions across all predefined subgroup analyses. Between-subgroup heterogeneity was low for baseline renal function (χ^2^ = 1.42, df = 1, *P* = 0.23; *I*^2^ = 29.3%) and for CIN definitions (χ^2^ = 2.72, df = 2, *P* = 0.26; *I*^2^ = 26.6%), and absent for diabetes status, the timing of post-procedure creatinine assessment, RIPC cycles, and cuff pressure strategy. Although moderate between-subgroup heterogeneity was suggested for procedural type, the subgroup difference was not statistically significant (χ^2^ = 2.22, df = 1, *P* = 0.14; *I*^2^ = 55.0%).

### Serum creatinine

Change in serum creatinine was reported in seven trials. Compared with the control group, RIPC was associated with a statistically significant attenuation of the postprocedural increase in serum creatinine (SMD −1.00, 95% CI −1.60 to −0.39, *P* = 0.001). However, substantial between-study heterogeneity was observed (*I*^2^ = 94%; Supplementary Figure S3A). Post-procedural serum creatinine levels were reported in 12 trials. Similarly, post-procedural serum creatinine levels were significantly lower in the RIPC group than in the control group (SMD −0.23, 95% CI −0.39 to −0.08, *P* = 0.003) (Supplementary Figure S3B).

### Estimated glomerular filtration rate

Post-procedural estimated glomerular filtration rate (eGFR) was reported in six trials. The pooled analysis showed no significant difference in post-procedural eGFR between the RIPC and control groups (SMD 0.11, 95% CI −0.03 to 0.25, *P* = 0.13) (Supplementary Figure S4).

### Neutrophil gelatinase-associated lipocalin

Post-procedural neutrophil gelatinase-associated lipocalin (NGAL) levels were reported in three trials. Compared with the control group, RIPC was associated with a borderline reduction in post-procedural NGAL levels, although the difference did not reach statistical significance (SMD −0.24, 95% CI −0.49 to 0.00, *P* = 0.05) (Supplementary Figure S5).

### Cystatin C

Post-procedural cystatin C levels were reported in five trials. Compared with the control group, RIPC was associated with numerically lower post-procedural cystatin C levels, but this difference was not statistically significant (SMD −0.37, 95% CI −0.77 to 0.03, *P* = 0.07) (Supplementary Figure S6).

### Clinical outcomes

MAEs were reported in 11 trials. The pooled analysis showed that RIPC significantly reduced the risk of MAEs compared with the control group (RR 0.55, 95% CI 0.41–0.75, *P* = 0.0001) (Figure 3).

**Figure 3:**
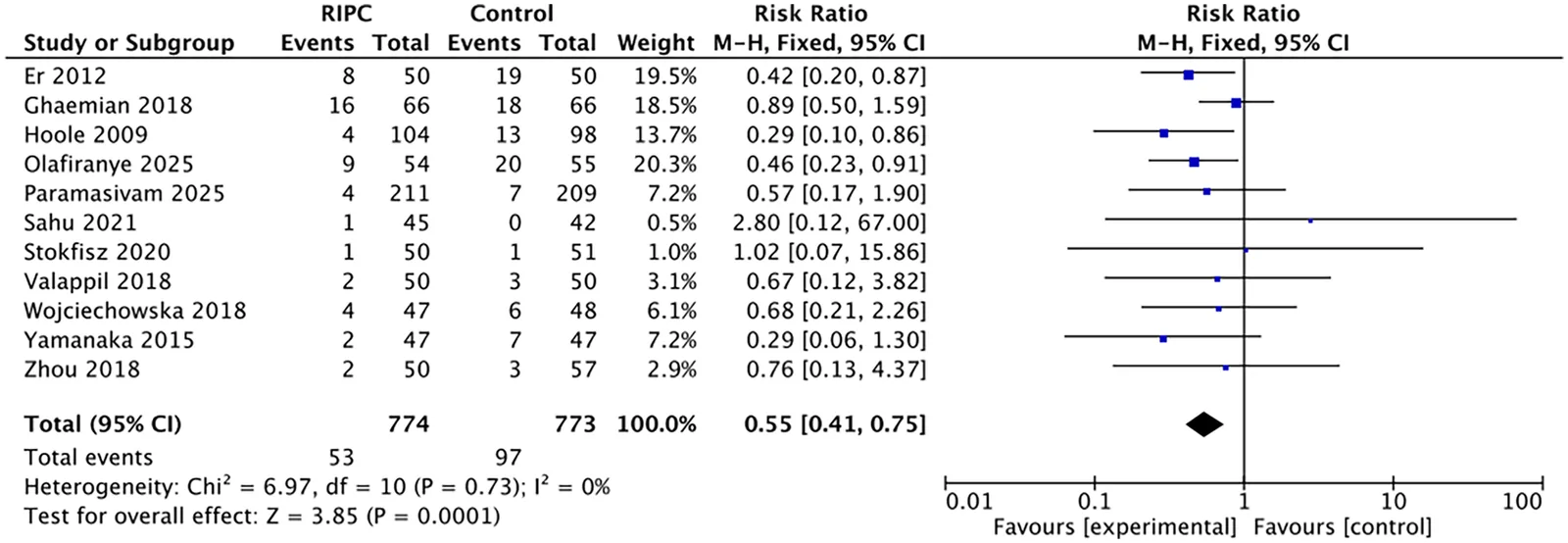
Effect of RIPC on MAEs.

In an exploratory subgroup analysis stratified by baseline eGFR, the association between RIPC and MAEs was not statistically significant in trials enrolling patients with eGFR < 60 mL/min/1.73 m^2^ (RR 0.82, 95% CI 0.50–1.35, *P* = 0.43), whereas a significant association was observed in trials enrolling patients with eGFR ≥ 60 mL/min/1.73 m^2^ (RR 0.44, 95% CI 0.30–0.65, *P* < 0.0001). The test for subgroup differences was borderline significant (χ^2^ = 3.81, df = 1, *P* = 0.05; *I*^2^ = 73.8%) (Supplementary Figure S7). This exploratory finding should therefore be interpreted cautiously.

All-cause mortality was reported in 11 trials. No significant difference in mortality was observed between the RIPC and control groups (RR 0.61, 95% CI 0.36–1.04, *P* = 0.07) (Supplementary Figure S8). The dialysis requirement was reported in 10 trials, and RIPC was not associated with a significant reduction in dialysis requirement (RR 0.82, 95% CI 0.33–2.05, *P* = 0.67) (Supplementary Figure S9). Rehospitalization was reported in 6 trials, with no significant difference observed between the RIPC and control groups (RR 0.83, 95% CI 0.62–1.12, *P* = 0.22) (Supplementary Figure S10).

### Publication bias and quality assessment

Visual inspection of the funnel plots showed no obvious asymmetry, suggesting no clear evidence of publication bias (Supplementary Figure S13). For outcomes with at least 10 included studies, Egger's regression test was further performed to assess potential small-study effects. The test did not indicate significant small-study effects for CIN (*P* = 0.30), serum creatinine levels (*P* = 0.24), or MAEs (*P* = 0.84) (Supplementary Table S5). Individual trial risk-of-bias assessments and GRADE certainty ratings are summarized in Supplementary Table S2. Most RCTs were judged to have a low overall risk of bias. According to the GRADE assessment, the certainty of evidence was rated as high for CIN, MAEs, and eGFR; moderate for all-cause mortality, dialysis requirement, rehospitalization, serum creatinine level, and NGAL; and low for change in serum creatinine from baseline and cystatin C.

### Trial sequential analysis

The TSA results are presented in Figures 4, 5, and Supplementary Figure S14. For CIN incidence, the cumulative Z-curve crossed both the conventional significance boundary and the trial sequential monitoring boundary and reached the required information size (RIS) of 3,893. For MAEs, the cumulative Z-curve crossed the conventional significance boundary and reached the RIS of 847 but did not cross the trial sequential monitoring boundary. For all-cause mortality, the cumulative Z-curve crossed the conventional significance boundary but did not cross the trial sequential monitoring boundary or reach the RIS of 5,567. For dialysis requirement, the cumulative Z-curve did not cross either the conventional significance boundary or the trial sequential monitoring boundary, and the RIS was not reached. For rehospitalization, the cumulative Z-curve initially crossed the conventional significance boundary during the early cumulative analyses but subsequently fell below it; an RIS of 5,980 would be required to draw a firm conclusion regarding this outcome.

**Figure 4:**
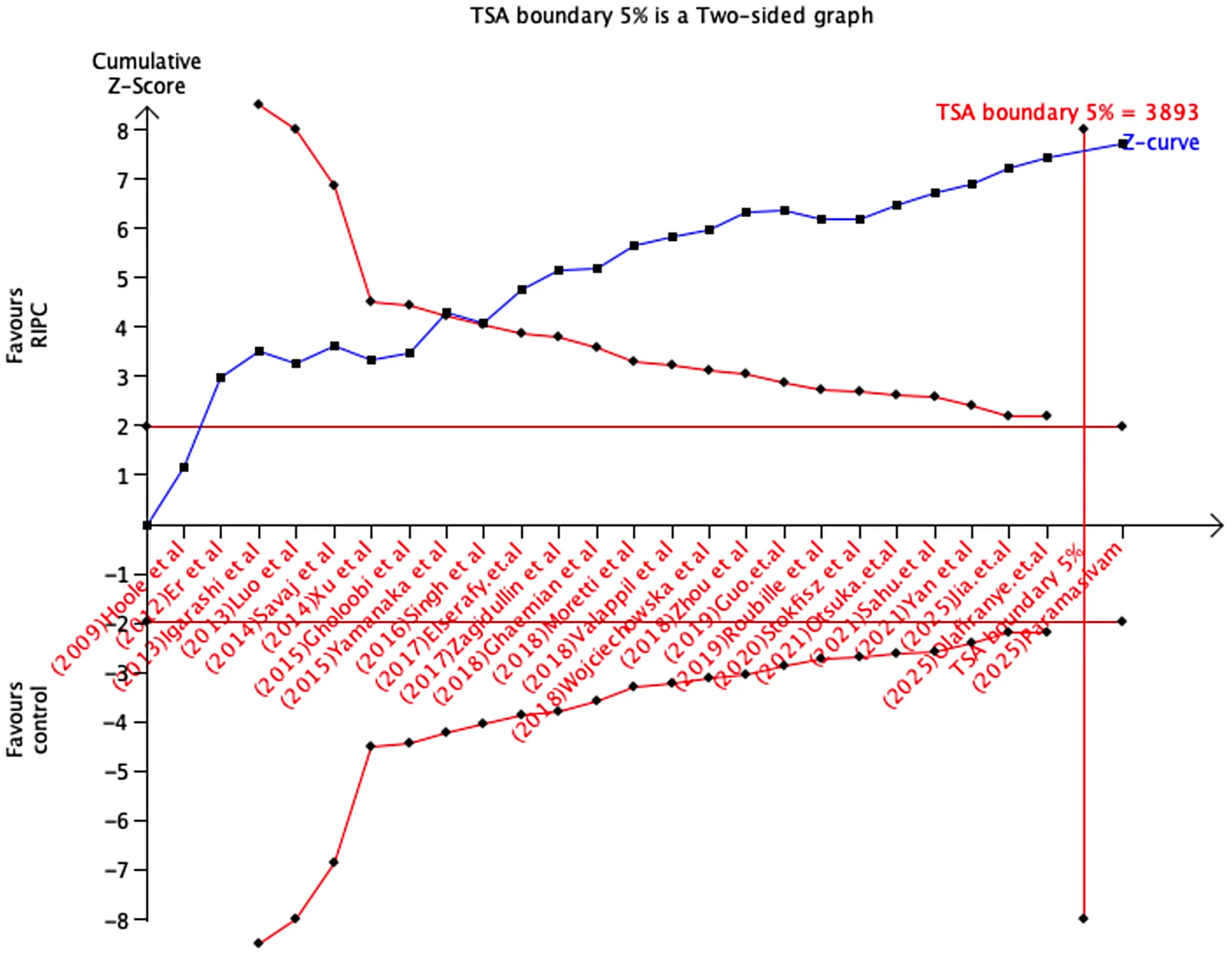
Trial sequential analysis of the effects of RIPC on CIN.

**Figure 5:**
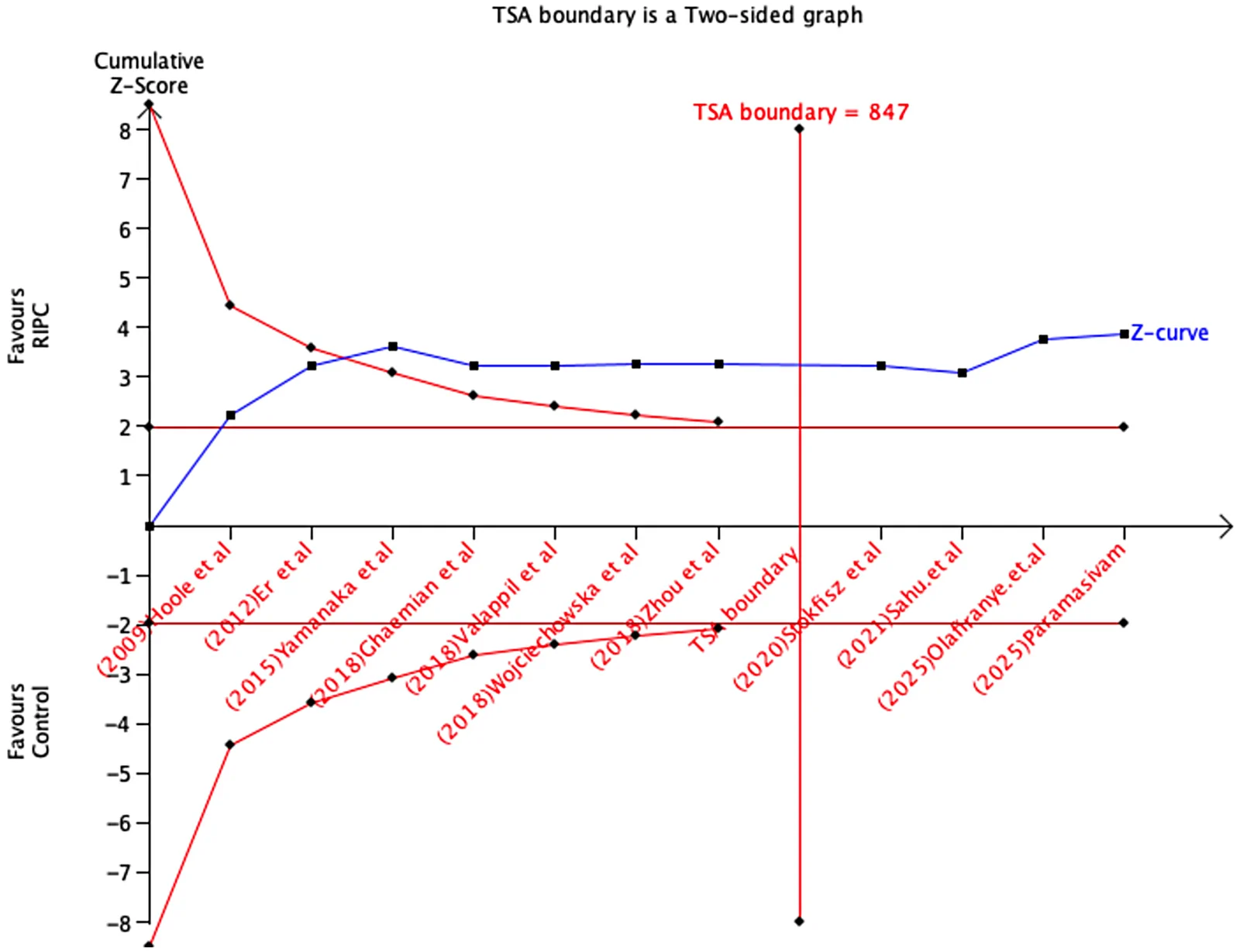
Trial sequential analysis of the effects of RIPC on MAEs.

## Discussion

RIPC was associated with a significantly lower risk of CIN and significantly fewer MAEs in patients undergoing coronary angiography or percutaneous coronary intervention, and it attenuated postprocedural increases in serum creatinine levels and was associated with lower postprocedural serum creatinine levels, while evidence for other renal biomarkers and clinical outcomes remains inconclusive.

CIN remains a clinically consequential complication after coronary angiography or PCI, contributing to prolonged hospitalization, increased healthcare costs, and adverse long-term outcomes (43, 44). The clinical burden is especially pronounced among elderly patients with multiple comorbidities who undergo PCI, as they are particularly susceptible to AKI (45). Current guidelines therefore emphasize AKI prevention before CA in such high-risk patients (46, 47). However, because conventional preventive strategies, including intravenous hydration, provide incomplete protection, simple adjunctive renoprotective strategies remain clinically relevant (48).

Previous randomized trials and meta-analyses have reported inconsistent or limited evidence regarding RIPC in CIN prevention. By incorporating updated randomized evidence and trial sequential analysis, the present study strengthens the evidence supporting a potential role of RIPC in CIN prevention and explores potential differences across predefined subgroups.

RIPC represents a noninvasive, low-cost intervention that can be readily applied before or during coronary procedures (11, 12). Nevertheless, variations in RIPC protocols, concomitant preventive regimens, and baseline patient characteristics should be considered when interpreting the pooled findings (49).

Current clinical guidelines for CIN prevention primarily recommend intravenous hydration as the cornerstone strategy, whereas RIPC is not routinely recommended because of inconsistent prior evidence (50). Our findings, showing an approximately 51% relative reduction in CIN risk and supported by trial sequential analysis, suggest that RIPC may warrant further consideration as an adjunctive preventive measure, particularly for patients at elevated renal risk undergoing coronary procedures.

The mechanisms responsible for RIPC-induced renal protection have not been fully characterized. Experimental and clinical studies indicate that RIPC may initiate remote organ-protective responses through circulating mediators and intracellular signaling cascades, including pathways related to nitric oxide and adenosine, danger signal–associated responses, attenuation of oxidative injury, and preservation of mitochondrial homeostasis. Through these coordinated effects, RIPC may enhance renal tolerance to ischemia–reperfusion-related injury (11, 51). This is consistent with the observed attenuation of postprocedural serum creatinine elevation in our analysis. However, this finding should be interpreted cautiously. The pooled analysis was based on only seven studies and demonstrated substantial between-study heterogeneity (*I*^2^ = 94%), indicating considerable inconsistency across trials. Moreover, the relatively wide confidence interval limits the precision of the pooled estimate. Therefore, although the result suggests a possible renoprotective effect of RIPC, it should not be regarded as definitive evidence of renal protection. No significant differences were detected in early renal biomarkers such as NGAL and cystatin C, and these null findings should be interpreted cautiously. These biomarkers differ from serum creatinine in their biological significance and temporal profiles, and inconsistent sampling times may have missed peak changes (52–54). Moreover, renal injury in the included populations was generally mild, and only a limited number of trials reported these outcomes, reducing the statistical power to detect modest effects (55). Similarly, the null findings for hard clinical endpoints, including all-cause mortality, dialysis requirement, and rehospitalization, may reflect low event rates and limited statistical power rather than a true absence of clinical benefit (56).

The timing of RIPC administration may also influence the activation and duration of these protective responses. Ischemic preconditioning is generally considered to involve two protective phases: an early phase that begins within minutes and lasts for several hours, and a delayed phase that emerges approximately 12–24 h after stimulation and may persist for several days (57, 58). In the present review, most RIPC protocols were initiated shortly before coronary angiography or PCI and therefore primarily reflected the early protective phase. The only included trial specifically evaluating a delayed RIPC strategy was conducted by Jia et al., who applied four cycles of 5-min upper-arm ischemia followed by 5 min reperfusion approximately 24 h before the procedure. Although this trial suggested a potential benefit of delayed RIPC in preventing CIN, only one study used this timing strategy. Therefore, the evidence remains insufficient to determine whether delayed RIPC is superior, equivalent, or mechanistically distinct from early RIPC. Accordingly, the current evidence base primarily reflects early RIPC protocols, whereas the potential clinical value and optimal implementation of delayed RIPC warrant further investigation.

Subgroup analyses may provide additional insights into patient selection. The protective association of RIPC against CIN was generally consistent across most predefined subgroups, including patients with diabetes mellitus, those undergoing PCI alone, and those undergoing mixed CA/PCI procedures. Importantly, this finding remained stable in analyses addressing heterogeneity in CIN definitions. The beneficial effect of RIPC persisted across CIN threshold categories, and the sensitivity analysis restricted to studies using ESUR-compatible definitions of CIN showed similar results, supporting the robustness of the observed benefit. Similarly, the association was consistent across different numbers of RIPC cycles and cuff pressure strategies, suggesting that the observed benefit was not confined to a specific RIPC cycle protocol or cuff pressure setting. Although the pooled estimate suggested a clearer association between RIPC and lower CIN incidence among studies enrolling patients with baseline eGFR <60 mL/min/1.73 m^2^, the interaction test did not provide statistical evidence of a differential effect across eGFR strata (P for subgroup differences = 0.23). The non-significant result in the eGFR ≥ 60 mL/min/1.73 m^2^ subgroup may reflect limited precision due to the small number of studies and sparse events. Thus, the apparent subgroup pattern is more consistent with a difference in precision than with robust evidence of effect modification. Accordingly, the eGFR-based subgroup analysis should be interpreted as exploratory and hypothesis-generating and does not justify concluding that RIPC is preferentially effective in patients with impaired renal function (59, 60). The MAEs subgroup findings should be interpreted cautiously. Their pattern was inconsistent with the CIN subgroup results and may reflect chance, limited power, study-level subgrouping, and heterogeneous MAE definitions across trials. Accordingly, these analyses should be viewed as exploratory rather than as evidence of effect modification (59, 60).

Deng et al. conducted a meta-analysis comparing RIPC with control for the prevention of CIN in patients undergoing CA or PCI, addressing a question closely related to ours (15). Their results showed that RIPC reduced the risk of CIN by approximately 50%, attenuated postprocedural increases in serum creatinine, and was associated with a borderline decrease in NGAL levels. Subgroup analyses according to baseline renal function and the timing of creatinine assessment yielded generally consistent findings. However, no significant differences were observed in eGFR, all-cause mortality, requirement for dialysis, or rehospitalization. Overall, these findings are largely consistent with our results. Compared with that prior meta-analysis, our study included an additional 10 RCTs, which may have improved the precision of the effect estimates and allowed a more comprehensive assessment of clinically relevant subgroups. Specifically, we performed additional subgroup analyses stratified by diabetes status and procedural type, as well as a pooled analysis of major adverse events. The renoprotective effect of RIPC was generally preserved across these additional analyses. Consistent with the prior meta-analysis, the benefit appeared more evident in patients with baseline eGFR < 60 mL/min/1.73 m^2^. These findings suggest that the renoprotective effect of RIPC may be relatively consistent across selected coronary procedural settings and may be particularly relevant in patients with pre-existing renal impairment. Therefore, targeted use of RIPC in moderate- to high-risk patients undergoing coronary procedures may represent a reasonable clinical strategy. After completion of our literature search, Wu et al. published a randomized controlled trial involving 484 patients with coronary heart disease undergoing elective PCI (61). The study reported a lower incidence of CI-AKI in the RIPC group than in the control group, which is consistent with the potential renoprotective effect observed in our meta-analysis. However, because this trial was published after the prespecified search cutoff date, it was not included in the quantitative synthesis. Future updates of this evidence base should incorporate this trial together with any other eligible studies published after our search cutoff date.

Notably, among the 26 included RCTs, 15 enrolled PCI-only cohorts, 5 included mixed CA/PCI populations, and only 6 focused on purely diagnostic coronary angiography. Because patients undergoing CA may proceed to PCI during the same procedure, purely diagnostic CA could not be consistently distinguished across trials. Therefore, procedural subgroup analyses compared PCI-only cohorts with CA or mixed CA/PCI cohorts. The renoprotective effect of RIPC did not differ materially between these subgroups. However, given the limited evidence from purely diagnostic CA, particularly in patients with preserved baseline renal function, the current findings should not be directly extrapolated to this population.

TSA indicated sufficient evidence to confirm a reduction in CIN risk with RIPC, suggesting that additional RCTs may not be necessary solely to verify this effect. However, the required information sizes for major adverse events, all-cause mortality, need for dialysis, and rehospitalization have not been reached. Therefore, current evidence remains insufficient to draw definitive conclusions regarding these outcomes.

## Conclusions

In patients undergoing coronary angiography or percutaneous coronary intervention, RIPC was associated with reduced risks of CIN and MAEs, attenuated postprocedural increases in serum creatinine levels, and lower postprocedural serum creatinine levels, while evidence for postprocedural eGFR, NGAL, cystatin C, and clinical outcomes remains inconclusive.

### Limitations

Despite the aforementioned strengths, several limitations should be acknowledged. First, the methodological quality of the included studies was variable, which may have affected the certainty of the evidence. Second, the definitions used for CIN and the definitions and components of MAEs varied across studies, potentially contributing to clinical heterogeneity. Third, variations in baseline characteristics, contrast media exposure, concomitant medications, hydration strategies, and RIPC protocols across studies may have influenced the observed treatment effects and limit the precision with which the pooled estimate can be interpreted. Fourth, several secondary renal outcomes were reported by a limited number of trials, limiting the precision of these estimates. Finally, most trials had relatively short follow-up durations, and some subgroup analyses included few studies; therefore, the effects of RIPC on long-term hard clinical outcomes and subgroup-specific findings should be interpreted cautiously.

## Data Availability

The original contributions presented in the study are included in the article/Supplementary Material. Further inquiries can be directed to the corresponding author.
