# Supplementary material for: Remote ischemic preconditioning reduces contrast-induced nephropathy in patients undergoing coronary angiography or percutaneous coronary intervention: an updated meta-analysis of randomized controlled trials
Source: Front Cardiovasc Med. 2026 Sep 18;13:1916701. doi: 10.3389/fcvm.2026.1916701 (PMC13630663; doi:10.3389/fcvm.2026.1916701)
Supplement: Supplementary file 1 [file Datasheet1.docx]

**Table S1. Search strategy used in each database searched**

| **Database** | **Search date** | **Complete search query** |
| --- | --- | --- |
| PubMed | Inception to March 1, 2026 | #1 "Ischemic Preconditioning"[MeSH Terms]  OR "remote ischemic precondition*"[tiab]  OR "remote ischaemic precondition*"[tiab]  OR "ischemic precondition*"[tiab]  OR "ischaemic precondition*"[tiab]  OR "ischemic condition*"[tiab]  OR RIPC[tiab]  #2 "Acute Kidney Injury"[MeSH Terms]  OR "contrast induced nephropath*"[tiab]  OR "contrast-induced nephropath*"[tiab]  OR "contrast-induced acute kidney injur*"[tiab]  OR CIN[tiab]  OR CI-AKI[tiab]  #3 "Randomized Controlled Trial"[pt]  OR "Controlled Clinical Trial"[pt]  OR (random*[tiab] AND trial[tiab])  #4 #1 AND #2 AND #3 |
| Embase | Inception to March 1, 2026 | #1 'ischemic preconditioning'/exp  #2 'ischemic conditioning'/exp  #3 'remote ischemic preconditioning'/exp  #4 remote ischemic precondition*:ti,ab OR remote ischaemic precondition*:ti,ab OR remote ischemic condition*:ti,ab OR remote ischaemic condition*:ti,ab OR remote precondition*:ti,ab OR RIPC:ti,ab  #5 ischemic precondition*:ti,ab OR ischaemic precondition*:ti,ab OR ischemic condition*:ti,ab OR ischaemic condition*:ti,ab  #6 #1 OR #2 OR #3 OR #4 OR #5  #7 'contrast induced nephropathy'/exp  #8 'acute kidney injury'/exp  #9 'acute kidney failure'/exp  #10 contrast induced nephropath*:ti,ab OR contrast-induced nephropath*:ti,ab OR contrast associated nephropath*:ti,ab OR contrast-associated nephropath*:ti,ab OR CIN:ti,ab  #11 contrast-induced acute kidney injur*:ti,ab OR contrast induced acute kidney injur*:ti,ab OR contrast-associated acute kidney injur*:ti,ab OR contrast associated acute kidney injur*:ti,ab OR CI-AKI:ti,ab OR CA-AKI:ti,ab  #12 acute kidney injur*:ti,ab OR acute renal injur*:ti,ab  #13 acute kidney failur*:ti,ab OR acute renal failur*:ti,ab OR acute kidney insufficien*:ti,ab OR acute renal insufficien*:ti,ab  #14 #7 OR #8 OR #9 OR #10 OR #11 OR #12 OR #13  #15 #6 AND #14 |
| Cochrane CENTRAL | Inception to March 1, 2026 | #1 MeSH descriptor: [Ischemic Preconditioning] explode all trees  #2 (remote AND (ischemic precondition* OR ischaemic precondition* OR ischemic condition* OR ischaemic condition* OR RIPC)):ti,ab,kw  #3 (ischemic precondition* OR ischaemic precondition* OR ischemic condition* OR ischaemic condition*):ti,ab,kw  #4 #1 OR #2 OR #3  #5 MeSH descriptor: [Acute Kidney Injury] explode all trees  #6 (contrast induced nephropath* OR contrast-induced nephropath* OR contrast nephropath* OR contrast media nephropath* OR CIN):ti,ab,kw  #7 (contrast-induced acute kidney injur* OR contrast induced acute kidney injur* OR contrast-associated acute kidney injur* OR contrast associated acute kidney injur* OR CI-AKI OR CA-AKI):ti,ab,kw  #8 (acute kidney injur* OR acute renal injur*):ti,ab,kw  #9 (acute kidney failur* OR acute renal failur*):ti,ab,kw  #10 (acute kidney insufficien* OR acute renal insufficien*):ti,ab,kw  #11 #5 OR #6 OR #7 OR #8 OR #9 OR #10  #12 #4 AND #11 |

**Figure S1. The results of Risk of bias.**

**A**

**B**

**C**

**D**

**E**

**F**

**G**

**Figure S2 (A-G)**. **Subgroup analysis of contrast-induced nephropathy according to predefined study-level characteristics.**

A. **baseline eGFR; B. Type of procedure; C. Diabetes status; D. Timing of CIN assessment; E. RIPC cycles; F. cuff pressure strategy; G. CIN definitions.**

**A**

**B**

**Figure S3 (A-B)**. **Effect of RIPC on Serum creatinine. A. Change in Serum creatinine; B. Post-procedural serum creatinine levels**

**Figure S4**. **Effect of RIPC on eGFR**

**Figure S5**. **Effect of RIPC on NGAL**

**Figure S6**. **Effect of RIPC on Cystatin C**

**Figure S7.** **Subgroup analysis of MAEs according to baseline eGFR**

**Figure S8.** **Effect of RIPC on All-cause mortality**

**Figure S9. Effect of RIPC on dialysis requirement**

**Figure S10. Effect of RIPC on Rehospitalization**

**Figure S11. Sensitivity analysis restricted to studies using ESUR-compatible definitions of CIN**

**Figure S12. Sensitivity analysis excluding the Jia et al. trial with a delayed-window RIPC strategy**

**Table S2. The result of and certainty of evidence assessment**

| **Certainty** **assessmen** | | | | | | |  | |  | |  |  |
| --- | --- | --- | --- | --- | --- | --- | --- | --- | --- | --- | --- | --- |
|  |  |  |  |  |  |  |  |  |  |  |  |  |

**Incidence** **of** **CIN**

| 25 | randomised  trials | not serious | not serious | not serious | not serious | none | 153/1997 (7 .7%) | 313/2010 (15 .6%) | **RR** **0.49**  (0 .41 to 0 .59) | **79** **fewer** **per**  **1,000**  (from 92 fewer  to 64 fewer) | ⨁⨁⨁⨁  High | CRITICAL |
| --- | --- | --- | --- | --- | --- | --- | --- | --- | --- | --- | --- | --- |

**MAEs**

| 11 | randomised  trials | not serious | not serious | not serious | not serious | none | 53/774 (6 .8%) | 97/773 (12 .5%) | **RR** **0.55**  (0 .41 to 0 .75) | **56** **fewer** **per**  **1,000**  (from 74 fewer  to 31 fewer) | ⨁⨁⨁⨁  High | CRITICAL |
| --- | --- | --- | --- | --- | --- | --- | --- | --- | --- | --- | --- | --- |

**All**-**cause** **mortailty**

| 11 | randomised  trials | not serious | not serious | not serious | seriousa | none | 19/1031 (1 .8%) | 33/1044 (3 .2%) | **RR** **0.61**  (0 .36 to 1 .04) | **12** **fewer** **per**  **1,000**  (from 20 fewer  to 1 more) | ⨁⨁⨁◯  Moderatea | CRITICAL |
| --- | --- | --- | --- | --- | --- | --- | --- | --- | --- | --- | --- | --- |

**dialysis** **requiramen**

| **t**  10 | randomised  trials | not serious | not serious | not serious | seriousb | none | 8/924 (0 .9%) | 10/934 (1 .1%) | **RR** **0.82**  (0 .33 to 2 .05) | **2** **fewer** **per**  **1,000**  (from 7 fewer  to 11 more) | ⨁⨁⨁◯  Moderateb | CRITICAL |
| --- | --- | --- | --- | --- | --- | --- | --- | --- | --- | --- | --- | --- |

**Rehospitalization**

| 6 | randomised  trials | not serious | not serious | not serious | seriousc | none | 67/559 (12 .0%) | 82/570 (14 .4%) | **RR** **0.83**  (0 .62 to 1 .12) | **24** **fewer** **per**  **1,000**  (from 55 fewer  to 17 more) | ⨁⨁⨁◯  Moderatec | CRITICAL |
| --- | --- | --- | --- | --- | --- | --- | --- | --- | --- | --- | --- | --- |

**Serum** **creatinine** **level**

| 12 | randomised  trials | not serious | seriousd | not serious | not serious | none | 702 | 713 | - | S MD **0.23** **SD**  **lower**  (0 .39 lower to  0 .08 lower) | ⨁⨁⨁◯  Moderated | IMPORTANT |
| --- | --- | --- | --- | --- | --- | --- | --- | --- | --- | --- | --- | --- |

**Change** **in** **Serum** **creatinine** **from** **baseline**

| 7 | randomised  trials | not serious | very seriouse | not serious | not serious | none | 402 | 401 | - | S MD **1** **SD**  **lower**  (1 .6 lower to  0 .39 lower) | ⨁⨁◯◯  Lowe | IMPORTANT |
| --- | --- | --- | --- | --- | --- | --- | --- | --- | --- | --- | --- | --- |

**eGFR**

| 6 | randomised  trials | not serious | not serious | not serious | not serious | none | 389 | 396 | - | S MD **0.11** **SD**  **higher**  (0 .03 lower to  0 .25 higher) | ⨁⨁⨁⨁  High | IMPORTANT |
| --- | --- | --- | --- | --- | --- | --- | --- | --- | --- | --- | --- | --- |

**NGAL**

| 3 | randomised  trials | not serious | not serious | not serious | seriousf | none | 127 | 133 | - | S MD **0.24** **SD**  **lower**  (0 .49 lower to  0 ) | ⨁⨁⨁◯  Moderatef | IMPORTANT |
| --- | --- | --- | --- | --- | --- | --- | --- | --- | --- | --- | --- | --- |

**Serum** **cystatin** **C**

| 5 | randomised  trials | not serious | seriousg | not serious | serioush | none | 310 | 313 | - | S MD **0.37** **SD**  **lower**  (0 .77 lower to  0 .03 higher) | ⨁⨁◯◯  Lowg,h | IMPORTANT |
| --- | --- | --- | --- | --- | --- | --- | --- | --- | --- | --- | --- | --- |

**CI:** confidence interval; **RR:** risk ratio; **SMD:** standardised mean difference **Explanations**

a . Downgraded one level for serious imprec ision because the confidence interval c rossed the line of no effect and the number of events was low.

b. Downgraded one level for serious imprec ision because of sparse events and a wide confidence interval c rossing the line of no effect .

c . Downgraded one level for serious imprec ision because the confidence interval c rossed the line of no effect and inc luded both apprec iable benefit and no c lear effect .

d. Downgraded one level for serious inconsistency due to moderate heterogeneity (I² = 50%) and variability in effect estimates ac ross studies .

e . Downgraded two levels for very serious inconsistency due to substantial heterogeneity (I² = 94%) , marked variability in effect estimates, and the presence of extreme outlying studies, resulting in an unstable pooled estimate .

f. Downgraded one level for serious imprec ision because the confidence interval reac hed the line of no effect and the sample siz e was small .

g. Downgraded one level for serious inconsistency due to substantial heterogeneity (I² = 81%) and considerable variability in effect estimates .

h. Downgraded one level for serious imprec ision because the confidence interval c rossed the line of no effect and inc luded both apprec iable benefit and no c lear effect .

**A	B**

**C	D**

**E**

**Figure S13 (A-E). Funnel plots.**

A. **CIN; B. serum creatinine levels; C. MAEs; D. All-cause mortality; E. dialysis requirement**

**A	B**

**C**

**Figure S14 (A-C).** **Trial sequential analysis of the effects of RIPC on All-cause mortality, Dialysis requirement, and Rehospitalization.A. All-cause mortality; B. Dialysis requirement; C. Rehospitalization.**

**Table S3. Classification of CIN definitions across included trials.**

| **Study** | **Simplified original definition** | **Threshold category** | **Time window category** |
| --- | --- | --- | --- |
| Hoole et al. 2009 | >25% at 24 h | ESUR-compatible | < 48h |
| Singh et al.2016 | ≥25% and/or ≥0.5 within 48 h | ESUR-compatible | ≥48h |
| Xu et al. 2014 | ≥25% and/or ≥0.5 at 16 h | ESUR-compatible | < 48h |
| Luo et al. 2013 | ≥25% and/or ≥0.5 at 16 h | ESUR-compatible | < 48h |
| Er et al. 2012 | ≥25% and/or ≥0.5 at 48 h | ESUR-compatible | ≥48h |
| Igarashi et al. 2013 | ≥25% and/or ≥0.5 within 48 h | ESUR-compatible | ≥48h |
| Savaj et al. 2014 | ≥30% and/or ≥0.3 at 24 h | Other | < 48h |
| Yamanaka et al. 2015 | ≥25% and/or ≥0.5 within 48–72 h | ESUR-compatible | ≥48h |
| Zagidullin et al. 2017 | ≥25% and/or ≥0.5 at 48 h | ESUR-compatible | ≥48h |
| Zhou et al. 2018 | ≥25% and/or ≥0.5 within 48–72 h | ESUR-compatible | ≥48h |
| Ghaemian et al. 2018 | ≥0.3 within 48–72 h | KDIGO-type | ≥48h |
| Moretti et al. 2018 | ≥25% and/or ≥0.5 within 48 h | ESUR-compatible | ≥48h |
| Valappil et al. 2018 | ≥25% and/or ≥0.5 within 24–48 h | ESUR-compatible | < 48h |
| Gholoobi et al. 2015 | ≥0.3 within 48 h | KDIGO-type | ≥48h |
| Wojciechowska et al. 2018 | ≥25% and/or ≥0.5 within 24–72 h | ESUR-compatible | ≥48h |
| Roubille et al. 2019 | ≥25% and/or ≥0.5 within 48–72 h | ESUR-compatible | ≥48h |
| Yan et al. 2021 | ≥25% and/or ≥0.5 within 48–72 h | ESUR-compatible | ≥48h |
| Stokﬁsz et al. 2020 | ≥25% and/or ≥0.5 within 48–72 h | ESUR-compatible | ≥48h |
| Sahu.et al. 2021 | ≥25% and/or ≥0.5 within 48 h | ESUR-compatible | ≥48h |
| Jia et al. 2025 | ≥0.3 within 48 h | KDIGO-type | ≥48h |
| Otsuka et al. 2021 | ≥20% and/or ≥0.3 within 24 h | Other | < 48h |
| Guo et al. 2019 | ≥25% and/or ≥0.5 within 48–72 h | ESUR-compatible | ≥48h |
| Elserafy et al. 2017 | ≥25% and/or ≥0.5 or CrCl decrease >50% within 48 h | Other | ≥48h |
| Bafna et al. 2020 | ≥25% and/or ≥0.5 within 48–72 h | ESUR-compatible | ≥48h |
| Olaﬁranye et al. 2025 | ≥50% and/or ≥0.3 at 48 h | KDIGO-type | ≥48h |
| Paramasivam et al. 2025 | ≥50% and/or ≥0.3 within 48 h | KDIGO-type | ≥48h |

CIN definitions were categorized according to the principal serum creatinine threshold reported in each trial. Definitions based on an increase in serum creatinine of ≥25% and/or ≥0.5 mg/dL were classified as ESUR; those based on an increase of ≥0.3 mg/dL or ≥50% were classified as KDIGO-type; and non-standard or mixed definitions were classified as Other. The post-contrast assessment window was recorded separately and was not used as the primary basis for categorization.

**Table S4. Hydration protocol reporting category**

| **Hydration protocol reporting category** | **Studies** |
| --- | --- |
| Complete or near-complete reported protocol | Wojciechowska 2018; Igarashi 2013; Yan 2021; Stokfisz 2020; Roubille 2019; Sahu 2021; Valappil 2018; Zhou 2018; Ghaemian 2018; Guo 2019; Paramasivam 2025; Bafna 2020; Jia 2025; Singh 2016; Er 2012 |
| Incomplete or non-standardized reported protocol | Zagidullin 2017; Moretti 2018; Olafiranye 2025; Savaj 2014; Gholoobi 2015; Yamanaka 2015; Elserafy 2017; Luo 2013 |
| No standardized prophylactic hydration protocol reported | Hoole 2009; Xu 2014; Otsuka 2021 |

**Table S5. Egger's test of CIN, serum creatinine levels, and MAEs。**

| **Outcomes** | **Number of studies** | **Egger’s test P value** |
| --- | --- | --- |
| CIN | 25 | 0.30 |
| serum creatinine levels | 12 | 0.24 |
| MAEs | 11 | 0.84 |
